# Patterns and predictors of exercise behavior during 24 months of follow-up after a supervised exercise program during breast cancer chemotherapy

**DOI:** 10.1186/s12966-020-00924-9

**Published:** 2020-02-14

**Authors:** Ki-Yong An, Dong-Woo Kang, Andria R. Morielli, Christine M. Friedenreich, Robert D. Reid, Donald C. McKenzie, Karen Gelmon, John R. Mackey, Kerry S. Courneya

**Affiliations:** 1 Faculty of Kinesiology, Sport, and Recreation, University of Alberta, Edmonton, Alberta T6G 2H9 Canada; 2 Alberta Health Services, Calgary, Alberta Canada; 3 University of Calgary, Calgary, Alberta Canada; 4 University of Ottawa Heart Institute, Ottawa, Ontario Canada; 5 University of British Columbia, Vancouver, British Columbia Canada; 6 British Columbia Cancer Agency, Vancouver, British Columbia Canada; 7 Cross Cancer Institute, Edmonton, Albera Canada

**Keywords:** Breast cancer, Cancer survivors, Exercise behavior, Exercise pattern, Physical activity, Determinants, Health-related fitness

## Abstract

**Background:**

Understanding the longer-term exercise behavior of patients with breast cancer after chemotherapy is important to promote sustained exercise. The purpose of the current study was to report the longer-term patterns and predictors of exercise behavior in patients with breast cancer who exercised during chemotherapy.

**Methods:**

In the Combined Aerobic and Resistance Exercise (CARE) Trial, 301 patients with breast cancer were randomized to three different exercise prescriptions during chemotherapy. Exercise behaviors after chemotherapy were self-reported at 6-, 12-, and 24-month follow-up. Exercise patterns were identified by categorizing patients according to which exercise guideline they were meeting (neither, aerobic only, resistance only, or combined) at each of the three follow-up timepoints (64 possible patterns). Predictors of longer-term exercise behavior included physical fitness, patient-reported outcomes, and motivational variables from the theory of planned behavior assessed at postintervention (postchemotherapy). Univariate and multivariate stepwise multinomial logistic regression and linear regression were used for statistical analyses.

**Results:**

A total of 264 (88%) participants completed all three follow-up exercise behavior assessments and exhibited 50 different exercise patterns. Postintervention aerobic fitness was the most consistent predictor of longer-term exercise behavior at all three timepoints. For example, higher aerobic fitness (per 1 ml/kg/min) predicted better adherence to the “aerobic only” (OR = 1.09; *p* = 0.005) and “combined” (OR = 1.12; *p* < 0.001) guidelines compared to “neither” guideline at 6-month follow-up. Additionally, higher postintervention muscular strength (per 1 kg) was associated with better adherence to the “resistance only” (OR = 1.07; *p* = 0.025) and “combined” (OR = 1.08; *p* < 0.001) guidelines compared to “neither” guideline at 24-month follow-up. Finally, lower perceived difficulty (per 1 scale point) was associated with better adherence to the “combined” (OR = 0.62; *p* = 0.010) and “aerobic only” (OR = 0.58; *p* = 0.002) guideline compared to the “neither” guideline at the 24-month follow-up.

**Conclusions:**

Our study is the first to show that the longer-term exercise patterns of patients with breast cancer who exercised during chemotherapy are diverse and predicted by physical fitness and motivational variables after chemotherapy. Our novel implications are that improving physical fitness during chemotherapy and applying motivational counseling after chemotherapy may improve longer-term exercise behavior in patients with breast cancer.

**Trial registration:**

(NCT00249015).

## Introduction

Exercise is physical activity that is planned, structured, repetitive, and purposive with the goal of improving or maintaining one or more components of physical fitness [1]. Exercise during breast cancer chemotherapy improves physical functioning [2], health-related fitness [2–5], treatment-related symptoms [2, 4, 5], self-esteem [3], sleep quality [6], chemotherapy completion rate [3, 5], and possibly even disease-specific and overall survival [7]. Unfortunately, many of the benefits of exercise during breast cancer chemotherapy dissipate quickly after cessation of the exercise program. Some studies have shown diminished effects as early as 6 months postintervention [5, 8–10] and few trials have even reported longer-term effects beyond 6 months [11–14]. We recently reported the 6-, 12-, and 24-month follow-up of the Combined Aerobic and Resistance Exercise (CARE) Trial which compared different types and doses of exercise during breast cancer chemotherapy [15]. We found that few of the short-term positive effects of the higher-dose aerobic and combined exercise programs during chemotherapy [2] were maintained during follow-up [15]. Conversely, there were strong positive associations between exercise behavior during the follow-up period and longer-term physical fitness, psychosocial outcomes, and quality of life [15]. These data suggest that longer-term maintenance of exercise after chemotherapy is critical for the sustained benefits of exercise.

Despite the importance of maintaining exercise after breast cancer chemotherapy, few studies have reported longer-term follow-up data on exercise behavior [11, 13, 14] and its determinants [14, 16, 17]. Moreover, these studies have reported mixed findings. One study reported no difference in exercise levels between the exercise and the control groups at 1-year follow-up [14] while some studies have reported higher physical activity in the exercise groups compared to the control groups even at four- or five-year follow-up [11, 13]. Findings on the determinants of exercise behavior regarding demographic, medical, motivational, and fitness variables have also been mixed [14, 16, 17]. Given that supervised exercise is emerging as a standard of care in this clinical setting, understanding exercise behavior and its determinants after a supervised exercise intervention during breast cancer chemotherapy is important to guide clinical and public health interventions.

The purpose of the current study was to report the longer-term exercise behavior patterns and predictors in survivors of breast cancer during the 24-month follow-up period in the CARE Trial. We included demographic and medical variables, patient-reported outcomes, health-related fitness outcomes, and motivational variables from the theory of planned behavior [18] as the candidate predictors based on previous findings [16, 17, 19–21]. We hypothesized that exercise participation rates would decline during follow-up and that few survivors of breast cancer would be meeting the combined aerobic and resistance exercise guidelines. Moreover, we hypothesized that younger age, better physical fitness, higher motivation, and lower fatigue would predict longer-term exercise behavior. Specifically, we hypothesized that higher aerobic fitness would predict greater adherence to the aerobic exercise guideline, while higher muscular strength and muscular endurance would predict better adherence to the resistance exercise guideline. The identification of exercise behavior patterns across the 6-, 12-, and 24-month follow-ups was considered exploratory.

## Methods

### Setting and participants

The methods of the CARE Trial have been reported elsewhere [2]. Briefly, the CARE Trial was a multicenter trial in Edmonton, Alberta; Ottawa, Ontario; and Vancouver, British Columbia. Ethics was approved for all three centers and written informed consent was obtained from all participants prior to trial enrollment. Participants were eligible for the study if they were women who could speak and understand English or French, were not pregnant, were aged 18 years or older, had stage I –IIIc breast cancer, and were initiating adjuvant chemotherapy. Women were excluded if they had incomplete axillary surgery, transverse rectus abdominis muscle reconstructive surgery, significant health problems, were not approved by their oncologist or, if they were performing structured vigorous-intensity exercise.

### Design and procedures

The study was a prospective, three-armed, randomized controlled trial. Patient-reported outcomes, including exercise behavior, were assessed at baseline, twice during chemotherapy, postintervention (postchemotherapy), and at 6-, 12-, and 24-month follow-up. Health-related fitness outcomes (i.e., body composition, aerobic fitness, and muscular fitness) were assessed at baseline, postintervention, and at the 12-month follow-up timepoint. After completing all baseline assessments, participants were stratified by center and chemotherapy protocol and randomly assigned in a 1:1:1 ratio to three exercise groups that performed a thrice-weekly (a) standard dose of 25–30 min of aerobic exercise (STAN), (b) a higher dose of 50–60 min of aerobic exercise (HIGH), or (c) a combined dose of 50–60 min of aerobic and resistance exercise (COMB).

### Exercise training intervention

The exercise training interventions have been described elsewhere [2]. Briefly, participants started the exercise intervention within 1–2 weeks of their first chemotherapy infusion and completed it within 3–4 weeks after their last chemotherapy infusion. The exercise programs were developed based on the Physical Activity Guidelines for Americans (USDHHS, [22], which have been endorsed for cancer survivors by the American College of Sports Medicine [4] and the American Cancer Society [23]. These guidelines recommend at least 75 min/week of vigorous-intensity aerobic exercise or 150 min/week of moderate-intensity aerobic exercise or any combination thereof. In the CARE trial, the STAN group was asked to follow the minimum physical activity recommendation and perform 75 min/week of vigorous-intensity aerobic exercise on either a cycle ergometer, treadmill, elliptical, rowing ergometer, or any combination of these modalities (i.e., 3 days/week for 25–30 min/session). The HIGH group was asked to double the minimum recommendation and perform 150 min/week of vigorous-intensity aerobic exercise (i.e., 3 days/week for 50–60 min/session). The COMB group was asked to follow the same aerobic exercise guidelines as the STAN group plus a standard resistance exercise program 3 days/week, consisting of two sets of 10–12 repetitions of nine different resistance exercises (e.g. leg extension, leg curl, leg press, calf raise, chest press, seated row, triceps extension, biceps curl, and modified curl-up) at 60–75% of their estimated one-repetition maximum (1RM) per session. The initial intensity of the aerobic exercise was individualized but generally began at 55–60% of peak oxygen consumption (VO_2peak_) and progressed to 70–75% of VO_2peak_ by week 6. Initial exercise duration was also individualized but generally began with sessions lasting 15–30 min and reached 25–30 min/session by week 4 (STAN and COMB) or 50–60 min/session by week 6 (HIGH). All exercise sessions were supervised by qualified exercise physiologists in the exercise facilities in or near the medical centers at each study location. The exercise intervention was performed only during chemotherapy which ranged from 12 to 18 weeks (median of 17 weeks). After the postintervention assessments, participants were given an exercise prescription for both aerobic and resistance exercise, however, they were free to do whatever exercise they chose and they received no further intervention.

### Assessment of exercise behavior during follow-up

Exercise behavior was assessed by a modified version of the Godin Leisure-Time Exercise Questionnaire [24] at baseline, 6-, 12-, and 24-month follow-up. The Godin Leisure-Time Exercise Questionnaire contains three questions that assess the average frequency of light intensity (e.g., easy walking, bowling), moderate-intensity (e.g., fast walking, folk dancing), and vigorous-intensity (e.g., running, cross-country skiing) exercise during free time in a typical week over the past month. We modified the questionnaire to include the average duration of exercise and to refer to a typical week over the past six months (6- and 12- month assessments) or over the past year (24- month assessment). We also included a separate question asking about the average duration and frequency of resistance exercise (e.g., use of free weights or universal equipment at home or at a fitness center). Participants were then categorized into four groups based on the American College of Sports Medicine [4] exercise guidelines for cancer survivors: (a) meeting “neither” exercise guideline; (b) meeting the “resistance only” guideline (i.e. ≥2 days/week of resistance exercise); (c) meeting the “aerobic only” guideline (i.e. ≥75 min/week of vigorous-intensity aerobic exercise or ≥ 150 min/week of moderate-intensity aerobic exercise or an equivalent combination); and (d) meeting the “combined” guidelines.

### Assessment of predictors

Exercise behavior predictors included non-modifiable factors such as demographic and medical variables, and modifiable factors such as patient-reported outcomes, health-related fitness outcomes, and Theory of Planned Behavior (TPB) variables based on previous findings showing several demographic [17, 19–21], motivational [16, 17], medical [16, 17], and health-related fitness [17] variables predicted exercise behavior in survivors of breast cancer. Demographic and health behavior information was collected at baseline by self-report and consisted of age (< 50 years versus ≥50 years), marital status (not married versus married), education (did not complete University/College versus completed University/College), annual family income (≥$80,000 versus <$80,000), employment status (unemployed versus employed), smoking status (non-smoker versus smoker), menopausal status (pre-menopausal versus peri and post-menopausal), group assignment (STAN versus HIGH versus COMB), and location/center (Ottawa versus Edmonton versus Vancouver).

Medical variables were collected from medical records and consisted of disease stage (I/IIa versus IIb/IIIa), type of surgery (lumpectomy versus mastectomy), and chemotherapy variables including chemotherapy type (i.e. taxane versus no taxane; anthracycline versus no anthracycline) and length of chemotherapy (i.e. 4 cycles (12 weeks) versus 6+ cycles (18+ weeks)).

Patient-reported outcomes were collected by self-report and consisted of the Medical Outcomes Survey Short Form (SF)–36 [25], Functional Assessment of Cancer Therapy-Breast (FACT-B) [26], FACT-Fatigue (FACT-F) [27], FACT-Endocrine Symptoms (FACT-ES) [28], FACT-Taxane [29], Perceived Stress Scale [30], revised Happiness Measure [31], Rosenberg Self-Esteem Scale [32], Center for Epidemiological Studies-Depression Scale (CES-D)-short form version [33–35], Spielberger State Anxiety Inventory [36], and Pittsburgh Sleep Quality Index (PSQI) [37, 38].

Health-related fitness assessments have been described elsewhere [2] and consisted of aerobic fitness (VO_2_peak) assessed by a maximal incremental exercise test on a treadmill; upper and lower body muscular strength and endurance assessed through chest and leg press tests; and body composition assessed using dual x-ray absorptiometry (DEXA).

TPB motivational variables were assessed by single items on a five-point scale [18] that have been commonly used in exercise and cancer survivor studies [17, 39–42]. Participants were asked to anticipate how beneficial, enjoyable, supported, motivated, and difficult they thought it would be to exercise over the next six months and if they had a detailed plan for where, when, and how they were going to exercise. Assessment of patient-reported outcomes, health-related fitness outcomes, and TPB variables at postintervention (postchemotherapy) were used for analyses.

### Statistical analyses

To ascertain the patterns of exercise behavior during the follow-up period, we identified the exercise behaviors (i.e., combined, aerobic only, resistance only, or neither) at each time point (i.e., 6 months, 12 months, and 24 months) and classified the exercise patterns based on all the possible combinations of exercise behavior spanning the three time points (4 × 4 × 4 = 64 possible patterns). For ease of interpretation, we then ignored the order of the exercise behaviors across the three timepoints and collapsed these 64 patterns into 14 possible patterns which included all the possible combinations of 1 (same exercise behavior at all 3 timepoints), 2 (2 different exercise behaviors during 3 timepoints), or 3 (a different exercise behavior at each timepoint). To analyze predictors across the three timepoints, we further categorized exercise patterns based on the number of times (0, 1, 2, or 3) “combined” or “neither” was reported in the exercise pattern.

To identify predictors of exercise behavior at each follow-up timepoint, we analyzed the univariate associations between the predictors and follow-up exercise behaviors using χ^2^ analyses for nominal variables and analyses of variance for continuous variables. Where possible, we dichotomized nominal variables based on standard groupings (e.g., age: < 50 versus ≥50 years; marital status: not married versus married; education level: did not complete University/college versus completed University/college). Health-related fitness outcomes, patient-reported outcomes, and motivational variables were analyzed as continuous variables with odds ratios (ORs) reported per whole unit of the measure (e.g., 1 ml/kg/min for aerobic fitness, 1 kg for the muscular strength measure, 1 repetition for the muscular endurance measure, 1 point on the 1–5 motivation scales, etc.). Predictor variables that had statistically significant univariate associations (*p* < 0.05) with exercise behaviors at each timepoint were further examined using a forward stepwise multinomial logistic regression to identify the main predictors of exercise behaviors at each timepoint. ORs (and inverse) of 1.68 (0.60), 3.47 (0.29), and 6.71 (0.15) are considered small, medium, and large effects, respectively [43].

We followed the same method to determine associations between predictors and exercise patterns based on the number of times “combined” or “neither” was reported in the exercise pattern (ranging from 0 to 3). We analyzed the univariate associations using χ^2^ analyses for nominal variables and analyses of variance for continuous variables. Predictor variables that had statistically significant univariate associations (*p* < 0.05) were examined in a linear regression model using the forward stepwise method to identify the main predictors of the “combined” and the “neither” exercise behaviors. Statistical analyses were performed using SPSS statistical software (IBM SPSS statistics 26).

## Results

**Fig. 1:**
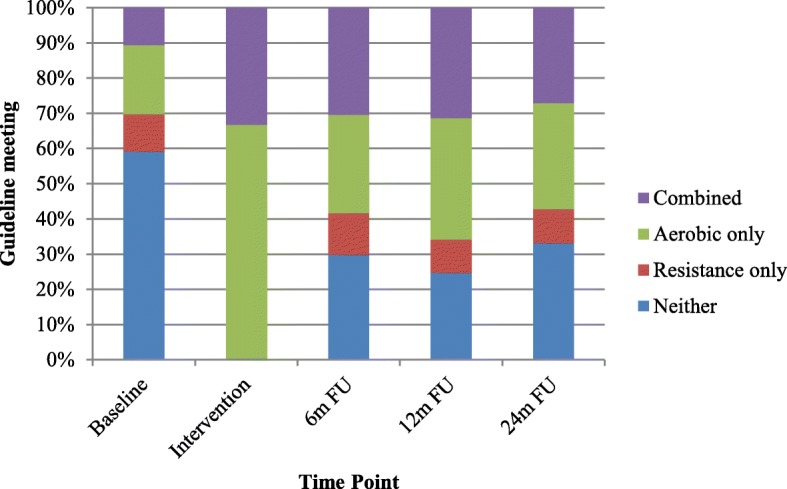
Proportion of participants meeting exercise guidelines. Note: intervention = randomized intervention period during chemotherapy

Flow of participants during the follow-up phase of the CARE Trial has been reported elsewhere [15]. Briefly, of the 301 randomized participants, 264 (88%) provided complete data at all three follow-up timepoints. Baseline characteristics of the CARE Trial participants have also been reported elsewhere [2]. For the 264 participants analyzed in the present study, the mean (and standard deviation) for age was 50.0 (8.7) years, and for BMI was 26.3 (5.5) kg/m2. For health-related fitness outcomes at postintervention, the mean (and standard deviation) for VO_2peak_ was 25.5 (5.9) ml/kg/min, for 1RM was 26.5 (9.0) kg for chest press and 87.6 (28.5) kg for leg press, for lean body mass was 41.3 (5.8) kg, and for body fat percent was 37.6 (8.5). For motivational variables at postintervention, the mean (and standard deviation) for TPB benefit was 4.9 (0.4), enjoyment was 4.1 (0.9), support was 4.6 (0.7), motivation was 4.4 (0.7), difficulty was 2.3 (1.0), and plan was 3.9 (1.1). The proportion of participants meeting neither, resistance only, aerobic only, and combined exercise guidelines at baseline, during the intervention (based on group assignment), and each of the three follow-up timepoints are presented in Fig. 1.

### Exercise patterns

**Fig. 2:**
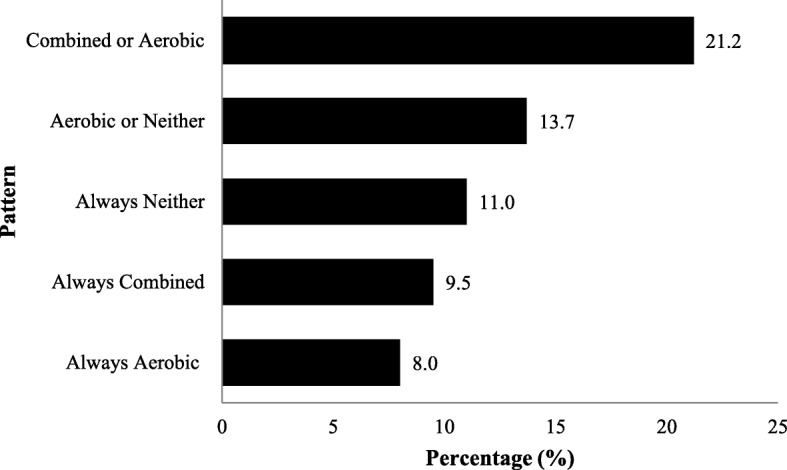
Most common exercise patterns of breast cancer patients during follow-up after exercise training during chemotherapy. Note: Aerobic = meeting aerobic exercise guideline only; Neither = meeting neither exercise guideline; Combined = meeting both exercise guidelines

Patients with breast cancer exhibited 50 out of a possible 64 exercise patterns across the three timepoints when including the temporal sequence of the exercise behavior. Of the 14 exercise patterns that ignored the temporal sequence, the most common patterns were “alternating combined or aerobic only” (21.2%), “alternating aerobic only or neither” (13.7%), “always neither” (11%), “always combined” (9.5%), and “always aerobic only” (8.0%), which accounted for 63.4% of all patterns (Table 1; Fig. 2). The percentage of participants who reported “combined exercise” 0, 1, 2, and 3 times during follow-up were 46.1%, 27.3%, 17.4%, and 9.5%, respectively. Similarly, the percentage of participants who reported “neither” 0, 1, 2, and 3 times during follow-up were 52.0%, 21.1%, 16.1%, and 11.0%, respectively.

**Table 1:** Exercise patterns across the three follow-up timepoints in breast cancer patients who exercised during chemotherapy

Pattern		N	%
Same exercise guideline at each of the three timepoints			
1	Neither	29	11.0
2	Combined	25	9.5
3	Aerobic only	21	8.0
4	Resistance only	4	1.5
Alternating two exercise guidelines across the three timepoints (in any order)			
5	Combined / Aerobic only	56	21.2
6	Aerobic only / Neither	36	13.7
7	Combined / Resistance only	19	7.2
8	Combined / Neither	19	7.2
9	Resistance only / Neither	17	6.5
10	Aerobic only / Resistance only	8	3.1
Different exercise guideline at each of the three timepoints (in any order)			
11	Combined / Aerobic only / Neither	16	6.0
12	Aerobic only / Resistance only / Neither	6	2.3
13	Combined / Resistance only / Neither	4	1.5
14	Combined / Aerobic only / Resistance only	4	1.5
Total		264	100

### Predictors of exercise behavior at 6-month follow-up

The significant univariate predictors of exercise behavior at 6-month follow-up were group assignment (*p =* 0.016), study site (*p* = 0.046), aerobic fitness (*p* < 0.001), upper body strength (*p* = 0.005), lower body strength (*p* = 0.035), lower body endurance (*p* = 0.038), fat mass (*p* = 0.007), body fat percent (*p* = 0.005), the Physical Component Summary domain of the SF-36 (*p* = 0.006), fatigue (*p* = 0.019), taxane symptom (*p* = 0.016), TPB enjoyment (*p* = 0.009), TPB support (*p* = 0.034), TPB motivation (*p* < 0.001), TPB difficulty (*p* = 0.003), and TPB planning (*p* < 0.001). The significant multivariate predictors of exercise behavior at 6-month follow-up were aerobic fitness, motivation, and planning (Table 2). Specifically, higher aerobic fitness (per 1 ml/kg/min) was associated with better adherence to the “aerobic only” (OR = 1.09; *p* = 0.005) and “combined” (OR = 1.12; p < 0.001) guidelines compared to “neither” guideline. Additionally, participants with higher aerobic fitness (per 1 ml/kg/min) were more likely to meet the “aerobic only” (OR = 1.10; *p* = 0.029) and “combined” (OR = 1.12; *p* = 0.006) guidelines when compared to the “resistance only” guideline. Moreover, participants were more likely to be meeting the “combined” guidelines compared to “neither” if they had higher motivation (OR = 2.03 per 1 point; *p* = 0.006) and a more specific plan (OR = 1.59 per 1 point; *p* = 0.008) for exercise postintervention.

**Table 2:** Stepwise multinomial logistic regression model estimating predictors of exercise behavior at 6-month follow-up (*n* = 263)

Step/predictors	Resistance vs. Neither		Aerobic vs. Neither		Combined vs. Neither	
	OR (95% CI)	*p*	OR (95% CI)	*p*	OR (95% CI)	*p*
1. VO_2peak_ (mL/kg∙min)	0.99 (0.91–1.08)	0.90	1.09 (1.03–1.16)	0.005	1.12 (1.05–1.19)	< 0.001
2. TPB Motivation (1-5)	1.25 (0.68–2.29)	0.47	1.47 (0.93–2.31)	0.10	2.03 (1.22–3.36)	0.006
3. TPB Plan (1-5)	1.51 (0.96–2.40)	0.08	1.28 (0.92–1.76)	0.14	1.59 (1.13–2.23)	0.008
	**Aerobic vs. Resistance**		**Combined vs. Aerobic**		**Combined vs. Resistance**	
	**OR (95% CI)**	***p***	**OR (95% CI)**	***p***	**OR (95% CI)**	***p***
1. VO_2peak_ (mL/kg∙min)	1.10 (1.01–1.19)	0.029	1.02 (0.97–1.08)	0.37	1.12 (1.03–1.22)	0.006
2. TPB Motivation [1–5]	1.17 (0.62–2.21)	0.62	1.38 (0.83–2.30)	0.22	1.62 (0.84–3.15)	0.15
3. TPB Plan [1–5]	0.84 (0.53–1.34)	0.47	1.25 (0.90–1.72)	0.18	1.05 (0.65–1.69)	0.84

Resistance = meeting resistance exercise guideline only; Aerobic = meeting aerobic exercise guideline only; Neither = meeting neither exercise guideline; Combined = meeting both exercise guidelines; TPB = theory of planned behavior, OR represents the change in the odds of a meeting a guideline for a one-unit change in the variables

### Predictors of exercise behavior at 12-month follow-up

The significant univariate predictors of exercise behavior at 12-month follow-up were age (*p* = 0.014), aerobic fitness (*p* < 0.001), upper body strength (*p* = 0.013), lower body strength (*p* = 0.028), lower body endurance (*p* = 0.020), body fat percent (*p* = 0.017), PCS (*p* < 0.001), sleep quality (*p* = 0.032), TPB motivation (*p* = 0.017) and TPB difficulty (*p* = 0.018). The significant multivariate predictors of exercise behavior at 12-month follow-up were aerobic fitness, sleep quality, difficulty, and lower body endurance (Table 3). Participants with higher aerobic fitness (per 1 ml/kg/min) were more likely to meet the “aerobic only” guideline (OR = 1.12; *p* < 0.001) when compared to “neither” guideline. Additionally, higher exercise difficulty (per 1 point) was associated with worse adherence to the “combined” guidelines compared to the “neither” guideline (OR = 0.60; *p* = 0.004), and participants with lower sleep quality (per 1 point) were more likely to meet the “combined” guidelines compared to the “aerobic only” (OR = 1.11; *p* = 0.005) and “resistance only” guideline (OR = 1.13; *p* = 0.049). Lastly, lower body endurance (per 1 repetition) predicted aerobic exercise (OR = 1.02; *p* = 0.043) and combined exercise (OR = 1.02; *p* = 0.015) compared to resistance exercise.

**Table 3:** Stepwise multinomial logistic regression model estimating predictors of exercise behavior at 12- month follow-up (n = 263)

Step/predictors	Resistance vs. Neither		Aerobic vs. Neither		Combined vs. Neither	
	OR (95% CI)	*p*	OR (95% CI)	*p*	OR (95% CI)	*p*
1. VO_2peak_ (mL/kg∙min)	1.04 (0.95–1.15)	0.38	1.12 (1.05–1.20)	< 0.001	1.07 (1.00–1.14)	0.05
2. TPB Difficulty (1–5) ^a^	0.91 (0.56–1.48)	0.72	0.80 (0.58–1.12)	0.19	0.60 (0.43–0.85)	0.004
3. Sleep quality (0–21) ^a^	0.93 (0.83–1.06)	0.27	0.95 (0.88–1.03)	0.23	1.05 (0.98–1.14)	0.19
4. lower body endurance (reps)	0.99 (0.97–1.00)	0.12	1.00 (1.00–1.01)	0.42	1.01 (1.00–1.02)	0.11
	**Aerobic vs. Resistance**		**Combined vs. Aerobic**		**Combined vs. Resistance**	
	**OR (95% CI)**	***p***	**OR (95% CI)**	***p***	**OR (95% CI)**	***p***
1. VO_2peak_ (mL/kg∙min)	1.08 (0.98–1.18)	0.11	0.95 (0.90–1.00)	0.06	1.02 (0.93–1.12)	0.64
2. TPB Difficulty (1–5) ^a^	0.88 (0.55–1.41)	0.59	0.75 (0.55–1.03)	0.07	0.66 (0.40–1.07)	0.09
3. Sleep quality (0–21) ^a^	1.02 (0.91–1.15)	0.73	1.11 (1.03–1.19)	0.005	1.13 (1.00–1.27)	0.049
4. lower body endurance (reps)	1.02 (1.00–1.04)	0.043	1.00 (1.00–1.01)	0.30	1.02 (1.00–1.04)	0.015

Resistance = meeting resistance exercise guideline only; Aerobic = meeting aerobic exercise guideline only; Neither = meeting neither exercise guideline; Combined = meeting both exercise guidelines; TPB = theory of planned behavior, OR represents the change in the odds of a meeting a guideline for a one-unit change in the variables

^a^ High scores indicate higher difficulty in exercise and worse sleep quality

### Predictors of exercise behavior at 24-month follow-up

The significant univariate predictors of exercise behavior at 24-month follow-up were study site (*p* = 0.049), aerobic fitness (*p* = 0.001), upper body strength (*p* < 0.001), lower body strength (*p* < 0.001), lower body endurance (*p* = 0.024), body fat percent (*p* = 0.016), taxane symptoms (*p* = 0.031), self-esteem (*p* = 0.026), TPB enjoyment (*p* = 0.017), TPB motivation (*p* = 0.001), TPB difficulty (*p* = 0.002), and TPB planning (*p* = 0.006). The significant multivariate predictors of exercise behavior at 24-month follow-up were upper body strength, difficulty, and aerobic fitness (Table 4). Higher upper body strength (per 1 kg) was associated with better adherence to the “combined” (OR = 1.08; *p* < 0.001) and “resistance only” (OR = 1.07; *p* = 0.025) guidelines compared to the “neither” guideline; and the “combined” guideline (OR = 1.05; *p* = 0.023) compared to the “aerobic only” guideline. Participants with lower exercise difficulty (per 1 point) were more likely to meet the “aerobic only” (OR = 0.58; *p* = 0.002) and the “combined” guideline (OR = 0.62; *p* = 0.010) compared to “neither” guideline. Moreover, higher aerobic fitness (per 1 ml/kg/min) was associated with better adherence to the “aerobic only” (OR = 1.09; *p* = 0.004) and the “combined” guidelines (OR = 1.07; *p* = 0.031) compared to “neither” guideline.

**Table 4:** Stepwise multinomial logistic regression model estimating predictors of exercise behavior at 24-month follow-up (*n* = 248)

Step/predictors	Resistance vs. Neither		Aerobic vs. Neither		Combined vs. Neither	
	OR (95% CI)	*p*	OR (95% CI)	*p*	OR (95% CI)	*p*
1. Upper body strength (kg)	1.07 (1.01–1.13)	0.025	1.04 (0.99–1.08)	0.10	1.08 (1.04–1.13)	< 0.001
2. TPB Difficulty (1–5) ^a^	0.86 (0.53–1.40)	0.54	0.58 (0.41–0.82)	0.002	0.62 (0.43–0.89)	0.010
3. VO_2peak_ (mL/kg∙min)	1.01 (0.93–1.11)	0.77	1.09 (1.03–1.16)	0.004	1.07 (1.01–1.14)	0.031
	**Aerobic vs. Resistance**		**Combined vs. Aerobic**		**Combined vs. Resistance**	
	**OR (95% CI)**	***p***	**OR (95% CI)**	***p***	**OR (95% CI)**	***p***
1. Upper body strength (kg)	0.97 (0.92–1.03)	0.28	1.05 (1.01–1.09)	0.023	1.02 (0.96–1.07)	0.58
2. TPB Difficulty [1–5] ^a^	0.67 (0.41–1.10)	0.11	1.08 (0.76–1.54)	0.67	0.72 (0.44–1.19)	0.20
3. VO_2peak_ (mL/kg∙min)	1.08 (0.99–1.17)	0.10	0.98 (0.93–1.04)	0.52	1.06 (0.97–1.15)	0.22

Resistance = meeting resistance exercise guideline only; Aerobic = meeting aerobic exercise guideline only; Neither = meeting neither exercise guideline; Combined = meeting both exercise guidelines; TPB = theory of planned behavior, OR represents the change in the odds of a meeting a guideline for a one-unit change in the variables

^a^ High scores indicate higher difficulty in exercise

### Predictors of exercise pattern during follow-up

Table 5 presents the predictors of the frequency of meeting the “combined” guideline or “neither” guideline during follow-up. The significant univariate predictors of the frequency of meeting the “combined” guideline during follow-up were aerobic fitness (*p* = 0.035), upper body strength (*p* = 0.001), lower body strength (*p* = 0.002), lower body endurance (*p* = 0.014), fatigue (*p* = 0.017), TPB motivation (*p* < 0.001), TPB difficulty (*p* = 0.020), and TPB plan (*p* = 0.002). In terms of multivariate predictors, upper body strength (β =0.19; *p* = 0.003), motivation (β =0.17; *p* = 0.006), and lower body endurance (β =0.14; *p* = 0.030) were associated with the frequency of meeting the “combined” guideline during follow-up.

**Table 5:** Stepwise linear regression model estimating predictors of the frequency of meeting the “Combined” or “Neither” guideline during follow-up (*n* = 245)

	Number of Combined (0–3)		
Step/predictors	Unstandardized B (95% CI)	Standardized β	*p*
1. Upper body strength	0.02 (0.01 to 0.04)	0.19	0.003
2. TPB Motivation	0.23 (0.07 to 0.40)	0.17	0.006
3. Lower body endurance	0.00 (0.00 to 0.06)	0.14	0.030
	**Number of Neither (0–3)**		
	**Unstandardized B (95% CI)**	**Standardized β**	***p***
1. VO_2peak_	−0.03 (−0.05 to −0.01)	−0.20	0.001
2. TPB Difficulty ^a^	0.22 (0.11 to 0.34)	0.22	< 0.001
3. Lower body strength	−0.01 (− 0.01 to − 0.00)	−0.21	0.001
4. Site (Edmonton vs. Ottawa/Vancouver)	−0.33 (− 0.58 to − 0.08)	−0.16	0.009

TPB = theory of planned behavior

^a^High score indicates higher difficulty in exercise

The significant univariate predictors of the frequency of meeting “neither” guideline during follow-up were study site (*p* = 0.020), aerobic fitness (*p* < 0.001), upper body strength (*p* = 0.002), lower body strength (*p* = 0.003), fat mass (*p* = 0.037), body fat percent (*p* = 0.017), the Physical Component Summary domain of the SF-36 (*p* = 0.009), TPB enjoyment (*p* = 0.007), TPB motivation (*p* < 0.001), TPB difficulty (*p* < 0.001), and TPB planning (*p* = 0.005). In terms of multivariate predictors, aerobic fitness (β = − 0.20; *p* = 0.001), exercise difficulty (β =0.22; *p* < 0.001), lower body strength (β = − 0.21; *p* = 0.001), and study site (Edmonton versus Ottawa/Vancouver) (β = − 0.16; *p* = 0.009) were associated with the frequency of meeting “neither” guideline during follow-up.

## Discussion

The purpose of the current study was to report the patterns and predictors of exercise behavior during 24 months of follow-up in patients with breast cancer who exercised during chemotherapy. The exercise patterns in our findings showed that patients with breast cancer exercised more during follow-up than at baseline but still showed a decline from the intervention period. The decline appeared immediately at 6-month follow-up and then remained stable at 12- and 24-month follow-up. Previous studies examining exercise interventions during breast cancer treatment have shown similar results. Husebo et al. [44] reported that physical activity levels at posttreatment and at 6-month follow-up were higher than baseline even after a home-based intervention. Mutrie et al. [8] also reported that patients with breast cancer increased their leisure-time physical activity after a supervised intervention during treatment but it was not maintained at 6-month follow-up, although it was still higher than baseline. Additionally, Schmidt et al.’s study [14] showed that resistance training during treatment enhanced participation in resistance exercise after treatment, but the effect attenuated in the longer-term follow-up. These results suggest that exercise training during chemotherapy may help patients with breast cancer improve longer-term exercise behavior compared to baseline; however, additional interventions may be needed to sustain the exercise program that was initiated during chemotherapy.

A novel finding of our study is that the longer-term exercise patterns of patients with breast cancer who exercised during chemotherapy are diverse and unstable with 50 out of a possible 64 exercise patterns being exhibited. Moreover, only 30% of participants reported a stable exercise pattern (including no exercise) across all three timepoints. Ignoring the temporal sequence of the exercise patterns, the most common exercise patterns were: “alternating combined or aerobic only”, “alternating aerobic only or neither”, “always neither”, “always combined”, and “always aerobic” with over 60% of participants falling into these 5 patterns. The two most common exercise patterns appear to identify groups of patients with breast cancer who (a) are trying to add resistance exercise to aerobic exercise and (b) are trying to maintain aerobic exercise versus no exercise.

Few studies to date have reported exercise behavior patterns in cancer survivors because they have not assessed exercise behavior at multiple timepoints and/or they have not assessed aerobic and resistance exercise separately. Courneya and Friedenreich examined exercise patterns by asking colorectal [45] and breast [46] cancer survivors to retrospectively report their exercise behavior prediagnosis, during active treatment, and posttreatment. Both studies reported four main exercise patterns including maintainers (active-active-active), temporary relapsers (active-inactive-active), permanent relapsers (active-inactive-inactive), and nonexercisers (inactive-inactive-inactive). Limitations of these studies included the retrospective design and the failure to assess aerobic and resistance exercise separately. Our study is the first study to report exercise patterns prospectively with multiple follow-up timepoints after chemotherapy and obtain separate assessments for the major exercise modalities. Our data indicate that exercise behavior patterns after exercising during breast cancer chemotherapy are highly variable and unstable. Consequently, identifying the key predictors of longer-term exercise behavior patterns is important to inform possible interventions to promote exercise after chemotherapy.

Interestingly, physical fitness was the most consistent predictor of exercise behavior in patients with breast cancer after chemotherapy. Although physical fitness is a predictor of exercise behavior in several populations [47, 48], few studies have examined it as a predictor of exercise behavior after breast cancer chemotherapy. Courneya et al. [17] reported strength improvements and postintervention body mass index predicted 6-month follow-up exercise behavior in patients with breast cancer after chemotherapy. Meanwhile, Schmidt et al. [14] reported that prediagnosis exercise levels predicted exercise behaviors at the 12-month follow-up but muscle strength and VO_2peak_ did not. In our findings, aerobic fitness was the most important predictor of exercise behaviors, especially aerobic exercise. Aerobic fitness (VO_2peak_) predicted the inclusion of aerobic exercise in almost all exercise comparisons (e.g., aerobic versus neither, combined versus neither, combined versus resistance, number of neither) at almost all timepoints. Muscular fitness (endurance and strength) also predicted exercise behaviors, especially resistance exercise. Muscular fitness predicted the inclusion of resistance exercise behavior in multiple comparisons (e.g., resistance versus neither, combined versus neither, number of combined) at various timepoints. It seems clear that physical fitness after chemotherapy is a key predictor of longer-term exercise behavior in patients with breast cancer. These findings suggest that exercise programs that improve, or at least maintain, physical fitness during breast cancer chemotherapy may also improve longer-term exercise adherence. Moreover, clinical exercise specialists may want to target patients with breast cancer with low physical fitness after chemotherapy for a more intensive exercise behavior change program.

In addition to physical fitness, TPB motivational variables (difficulty, motivation, and planning) also predicted exercise behaviors, especially in comparison to neither (e.g., aerobic versus neither, combined versus neither, number of neither). Motivational variables are strong predictors of exercise behavior in many cancer survivor populations [49, 50], however, few studies have examined motivational variables as predictors of exercise behavior after exercising during chemotherapy. Courneya et al. [17] reported that instrumental attitude (perceived benefits) predicted exercise behavior 6 months after chemotherapy and Emery et al. [16] reported that family support predicted exercise behavior in breast cancer survivors 5-years after treatment. Our findings showed that intention (motivation and planning) is associated with short-term exercise behavior whereas perceived behavioral control (difficulty) is associated with longer-term exercise behavior after chemotherapy in patients with breast cancer. These findings suggest that clinical exercise specialists should help patients with breast cancer stay motivated by emphasizing novel and important benefits, developing a specific plan, and reducing the perceived difficulty of continuing to exercise after breast cancer chemotherapy.

It is also instructive to know the many variables that were not key predictors of longer-term exercise behavior. Of all the patient-reported outcomes examined, only sleep quality predicted exercise behavior but it was inconsistent. Demographic variables were not related to longer-term exercise behavior in multivariate analyses. Moreover, medical variables did not predict exercise behaviors after chemotherapy even in univariate analyses in the current study. However, several previous studies reported medical variables including surgery type, disease stage, and the receipt of chemotherapy associated with exercise behavior after breast cancer chemotherapy [16, 17]. Whether or not medical variables are associated with long-term exercise behaviors in patients with breast cancer after chemotherapy is still unclear and should be explored further. Finally, age, group assignment, fat mass, percent body fat, physical component score, taxane and endocrine symptoms, self-esteem, anxiety, TPB enjoyment, and TPB support were associated with exercise behavior in univariate but not multivariate analyses. These variables may be associated with exercise behavior and should be considered candidate predictors in future studies.

Our study has important strengths and weaknesses. Strengths of the present study include the multiple longer-term (6-, 12-, and 24-month) follow-ups, the separate assessment of aerobic and resistance exercise, the large sample size, the comprehensive assessment of candidate predictors, the objective measures of physical fitness, and the excellent follow-up rate. Moreover, the current study design is clinically relevant because it attempts to predict follow-up exercise behavior in patients with breast cancer who received supervised exercised during chemotherapy, which is quickly becoming the standard of care. Additionally, the findings of this study have practical implications. All significant predictors in multivariate analyses were modifiable factors, which means that it is clearly possible to change these factors to improve longer-term exercise behavior. Limitations of the present study include the self-reported exercise behavior, the homogeneous sample which may limit the generalizability of the results, the collapse of exercise patterns ignoring temporal sequence due to too many patterns, and different sample sizes at each timepoint. Another limitation is that we did not assess the measurement properties of our single item assessments of the motivational variables in our study and recommend multi-item assessments for future studies.

In summary, we examined the patterns and predictors of longer-term exercise behavior in patients with breast cancer who exercised during chemotherapy. We found that patients with breast cancer exhibited varied and inconsistent exercise patterns across the three longer-term follow-ups. The most common exercise patterns included a group of patients with breast cancer who oscillated between combined exercise and aerobic exercise only, and another group that oscillated between aerobic exercise only and no exercise. Moreover, the most consistent predictors of longer-term exercise behavior were physical fitness and motivational variables, whereas patient-reported outcomes, demographic, and medical variables played a limited role. These predictors should be targeted to help patients with breast cancer maintain exercise after chemotherapy and into survivorship. More specifically, medical professionals and exercise specialists should assist patients with breast cancer to maintain their physical fitness during treatment and counsel them on overcoming barriers and developing a detailed plan to continue exercise after chemotherapy.

## Data Availability

The data that support the findings of this study are available from the corresponding author upon reasonable request.
